# Predictors of mortality and short-term physical and cognitive dependence in critically ill persons 75 years and older: a prospective cohort study

**DOI:** 10.1186/1477-7525-9-35

**Published:** 2011-05-16

**Authors:** Cédric Daubin, Stéphanie Chevalier, Amélie Séguin, Cathy Gaillard, Xavier Valette, Fabrice Prévost, Nicolas Terzi, Michel Ramakers, Jean-Jacques Parienti, Damien du Cheyron, Pierre Charbonneau

**Affiliations:** 1 Department of Medical Intensive Care, Avenue Côte de Nacre, Caen University Hospital, 14033 Caen Cedex, France; 2 Department of Biostatistics Clinical Research, Avenue Côte de Nacre, Caen University Hospital, 14033 Caen Cedex, France; 3 Inserm ERI 27, Caen University, 14033 Caen Cedex, France and EA 4497 Versailles-Saint Quentin en Yvelines University, 92380 Garches, France; 4 Iserm UMR-S 707, Paris, F-75012, Université Pierre Marie Curie-Paris 6, UMR-S 707, Paris, F-75012, France; 5 UPRES EA 2128, Caen University, 14033 Caen Cedex, France

**Keywords:** older persons, intensive care unit, mortality, functional autonomy, quality of life

## Abstract

**Background:**

The purpose of this study was to identify predictors of 3-month mortality in critically ill older persons under medical care and to assess the clinical impact of an ICU stay on physical and cognitive dependence and subjective health status in survivors.

**Methods:**

We conducted a prospective observational cohort study including all older persons 75 years and older consecutively admitted into ICU during a one-year period, except those admitted after cardiac arrest, All patients were followed for 3 months or until death. Comorbidities were assessed using the Charlson index and physical dependence was evaluated using the Katz index of Activity of Daily Living (ADL). Cognitive dependence was determined by a score based on the individual components of the Lawton index of Daily Living and subjective health status was evaluated using the Nottingham Health Profile (NHP) score.

**Results:**

One hundred patients were included in the analysis. The mean age was 79.3 ± 3.4 years. The median Charlson index was 6 [IQR, 4 to 7] and the mean ADL and cognitive scores were 5.4 ± 1.1 and 1.2 ± 1.4, respectively, corresponding to a population with a high level of comorbidities but low physical and cognitive dependence. Mortality was 61/100 (61%) at 3 months. In multivariate analysis only comorbidities assessed by the Charlson index [Adjusted Odds Ratio, 1.6; 95% CI, 1.2-2.2; *p *< 0.003] and the number of organ failures assessed by the SOFA score [Adjusted Odds Ratio, 2.5; 95% CI, 1.1-5.2; *p *< 0.02] were independently associated with 3-month mortality. All 22 patients needing renal support after Day 3 died. Compared with pre-admission, physical (*p *= 0.04), and cognitive (*p *= 0.62) dependence in survivors had changed very little at 3 months. In addition, the mean NHP score was 213.1 **± **132.8 at 3 months, suggesting an acceptable perception of their quality of life.

**Conclusions:**

In a selected population of non surgical patients 75 years and older, admission into the ICU is associated with a 3-month survival rate of 38% with little impact on physical and cognitive dependence and subjective health status. Nevertheless, a high comorbidity level (ie, Charlson index), multi-organ failure, and the need for extra-renal support at the early phase of intensive care could be considered as predictors of death.

## Background

In industrialized countries, the older population is expected to grow faster than any other age groups [International Data Base: World population information http://www.census.gov/ipc/www/ibd/worldpopinfo.html]. Therefore, the number of critically ill older persons requiring intensive care is likely to increase substantially in the near future [1]. However, clinicians are sometimes reluctant to provide intensive care to older persons because of their shorter life expectancy and their high hospital and long-term mortality, specifically for those who are being treated medically or who undergo unplanned surgery [2,3]. However, survivors consider their self-sufficiency and their long-term quality of life satisfactory or good after an ICU stay [2,4-8]. In this context, providing predictors of short-term mortality or of impairment of physical and cognitive status could be useful for identifying critically ill older persons who could benefit from intensive treatment. For clinicians, identifying these patients is essential, both for preventing suffering related to unnecessary treatments, and for ensuring optimal use of finite resources. However, studies that specifically focus on these topics are scarce.

The aim of this study is to identify risk factors associated with 3-month mortality after ICU admission in critically ill older persons and to assess the clinical impact of an ICU stay on physical and cognitive dependence and subjective health status in survivors.

## Materials and methods

### Setting and Patients

This prospective observational cohort study was performed in the medical intensive care unit at the University Hospital of Caen, France, between November 2006 and October 2007. During the 12-month study period, 657 patients were admitted to the ICU. All older persons 75 years and over (*n *= 125) consecutively admitted to the ICU were assessed for eligibility. Surgical patients (*n *= 8) or patients who were obviously moribund or comatose after cardiac arrest (*n*= 17) were excluded from the analysis. All patients included were followed for 3 months or until death.

As a further note, during the study period 70 older patients (>75 years) requiring medical care but considered as too ill to benefit from intensive care, were withheld from the ICU.

### Study Design

The study protocol was submitted to the local independent ethics committee. The ethical board deemed that approval was not necessary, given the observational nature of this prospective study. Thus, in accordance with French legislation at the time of the study, no informed consent was obtained from the patients.

The following data were collected at the time of ICU admission for each patient: gender, age, marital status, location of usual residence, body mass index, underlying disease according to the Charlson index [9], physical dependence and cognitive status one month prior to admission, assessed by the Katz index of Activity of Daily Living (ADL) [10] and a cognitive score based on the individual components of the Lawton index of Daily Living (IADL) [11], date of admission to the emergency department or acute care hospital wards, number of organ failures according to the Sequential Organ Failure Assessment (SOFA) and the SOFA score [12], severity of illness according to the Simplified Acute Physiologic Score II (SAPS II) [13], and the Acute Physiology and Chronic Health Evaluation (APACHE II) [14], need for ventilation or renal dialysis, and reasons for ICU admission.

During their ICU stay, the SOFA score, the number of organ failures, shock and need for ventilation or renal dialysis were sequentially reassessed at Day 3 and Day 7. The duration of mechanical ventilation, the ICU and hospital length of stay, decision to activate care withdrawal and the discharge destination, were also recorded. In addition, the ICU, hospital and 3-month mortalities were recorded. Moreover, all survivors were assessed by telephone interview for physical dependence and cognitive status and for the subjective perception of social and personal effects of ICU stay using the Nottingham Health Profile (NHP) score [15], at 3 months following ICU admission.

### Definifions

The Charlson comorbidity index is based on the assignment of comorbidities observed in patients to one of several categories. A weighted score is assigned to each comorbidity, based on the relative risk of 1-year mortality. The sum of the index score is an indicator of disease burden and a predictor of death [9]. According to the modified version of the Charlson comorbidity index (applicable to the tenth revision of the International Classification of Diseases), 3 levels of comorbidity are defined: low (score = 0 or 1), medium (score = 2 to 4), and high (score = 5 or over) [16-18].

The Katz index of Activity of Daily Living (ADL) [10] assesses the ability of patients to perform the daily activities of bathing, dressing, toileting, transferring, continence and feeding. This index correlates with physical dependence. In this study, patient dependence was described in one of 2 manners for each function: independent (1 point), and dependent (0 points). The worst ADL score obtained was 0 (complete dependence) and the best was 6 (complete independence).

The cognitive score includes the individual components of the Lawton index of Daily Living: ability to handle finances, responsibility for own medications, ability to use the telephone and mode of transportation. This score correlates with impairment of cognitive functions independent of age, sex and education [11]. For each function, patient dependence is described in 2 degrees: not dependent (0 point), and dependent (1 point). The worst score obtained in this study was 4 (complete dependence) and the best 0 (complete independence).

The Nottingham Health Profile (NHP), used in its validated French version [15], assesses subjective health status by investigating the patient's subjective perception of social and personal effects of illness. It computes 38 statements divided into 6 categories: energy (3 questions), pain (8 questions), emotional reaction (9 questions), sleep (5 questions), social isolation (5 questions) and physical mobility (8 questions). In our study, the patients answered each question with "yes" (if there was a handicap, computed as 1) or "no" (if there was no handicap, computed as 0) about his/her situation at the time of the phone interview. Each "yes" was weighed according to its importance in the category and scored between 0 (maximum quality) and 100 (no quality). In each category, the worst score obtained was 100 and the best 0. The aggregate sum varied between 600 (maximum handicap) and 0 (no handicap). When a patient could not answer, the NPH score was not evaluated.

#### Statistical Analysis

Quantitative variables were expressed as means ± standard deviation or as the median associated with the Inter-Quartile range (IQR) when applicable. Qualitative variables were expressed as percentages. Firstly, we used logistic regression to analyze risk factors for mortality at 3 months for baseline patient characteristics at the time of ICU admission, and also to analyze clinical data during their ICU stay. Secondly, we constructed a multivariate model predicting the probability of mortality at 3 months by performing a stepwise logistic regression using baseline risk factors at the time of ICU admission. The Raw Odds Ratio (ROR) and the Adjusted Odds Ratio (AOR) are given with 95% Confidence Intervals (CI). A paired Student's *t*-test was used to compare physical dependence and cognitive status between pre-admission and the third month of follow-up. We used SPSS version 15.0 (Chicago, IL, USA) for data analysis. All tests were 2-sided and a *p*-value < 0.05 was considered statistically significant.

## Results

### Baseline Characteristics

**Figure 1:**
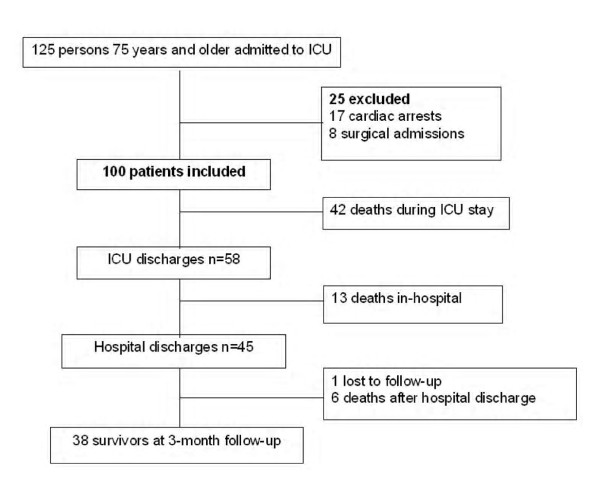
**Study profile**.

One hundred patients (65 male and 35 female) fulfilled the inclusion criteria for analysis. At 3 months, 61 patients (61%) had died (Figure 1). Baseline characteristics of admitted patients are shown in Table 1. The sex ratio (M/F) was 2/1. The mean age was 79.3 ± 3.4 years. Sixty-one patients were under 80 years old, 34 ranged from 80 to 85 years, and 5 were over 85. All patients but 9 lived at home, 58% of whom had been living with a partner before admission. The mean BMI was 27.3 ± 5.8, but 30 patients (30%) were obese (BMI >30). The median Charlson index was 6 [IQR, 4 to 7] and the mean physical dependence and cognitive scores were 5.4 ± 1.1 and 1.2 ± 1.4, respectively, corresponding to a population with a high level of comorbidities but low physical and cognitive dependence. According to the ADL index and cognitive score, respectively, 57% and 40% of the patients were completely independent (ADL index = 6, cognitive score = 0) and only 1% and 7% were completely dependent (ADL index = 0, cognitive score = 4). On ICU admission, the median SAPS II score and APACHE II score was 53 [IQR, 39 to 68] and 24 [IQR, 18 to 30], respectively. The main reasons for admission were respiratory disease (48%), cardiac disease (20%) and neurologic disease (12%). The median SOFA score was 7 [IQR, 5 to 7], and 24% of the patients satisfied multi-organ failure criteria (≥3 organ failures). With the exception of 12 patients, all required ventilator support; non invasive ventilation (NIV) in 25 patients (25%), and invasive mechanical ventilation in 63 patients (63%), 6 of whom received invasive mechanical ventilation after NIV failure. Forty-one patients (41%) were in shock and 12 patients (12%) needed additional renal support.

**Table 1:** Baseline characteristics of patients

Characteristics	Patients
Age (yrs), mean ± SD	79.3 ± 3.4

Male, n (%)	65 (65%)

BMI, mean ± SD	27.3 ± 5.8

Charlson index, median (IQR)	6 (4-7)
Low comorbidity level: score = 0 or 1	0
Medium comorbidity level: score = 2 to 4	28
High comorbidity level: score = 5 or over	71

ADL index, mean ± SD	5.4 ± 1.1

Cognitive score, mean ± SD	1.2 ± 1.4

Admission from, *n *(%)	
Emergency unit	54 (54%)
Medical unit	46 (46%)

Reason for admission, n (%)	
Cardiac disease	20 (20%)
Acute myocardial infarction	12
Acute pulmonary edema	7
Limb ischemia	1
Respiratory disease	48 (48%)
Pneumonia	24
Exacerbation of chronic obstructive disease	12
Exacerbation of chronic restrictive disease	6
Lung cancer	4
Pulmonary thrombosis	1
Quincke edema	1
Neurologic disease	12 (12%)
Acute stroke	6
Brain tumor	1
Meningitis	1
Epilepsy	1
Cerebral trauma	1
Amyotrophic lateral sclerosis	1
Tetanus	1
Abdominal disease	7 (7%)
Acute pancreatitis	3
Cirrhosis	2
Occlusive syndrome	2
Others	
Acute renal failure	3
Intoxication	3
Rhabdomyolysis	1
Unknown	6

SAPS II score, median (IQR)	53 (39-68)
APACHE II score, median (IQR)	24 (18-30)

SOFA score, median (IQR)	7 (5-10)

Organ failures, mean ± SD	1.4**±**1.2
≥3 organ failures, n (%)	24 (24%)

Assisted ventilation	
NIV	31 (31%)
MV	63 (63%)

Shock, n (%)	41 (41%)
Cardiogenic	15
Septic	22
Hemorragic	4

Extra-renal support initiated in ICU, n (%)	12 (12%)

BMI, body mass index; ADL, Activity of Daily Living; SAPS II score, Simpligfied Acute Physiologic Score II, APACHE II score, Acute Physiology and Chronic Health Evaluation; SOFA score, Sequential Organ Failure Assessment; NIV, non invasive ventilation; MV, mechanical ventilation.

### Risk Factors Associated with Mortality at 3 Months

At 3 months 61 patients (61%) had died: 42 during their ICU stay, 13 after ICU discharge, and 6 after hospital discharge. Therefore, the majority of non survivors died during the ICU stay, half of them in the first week.

Thirty-six patients were subject to treatment limitation decisions. Thirty two died. However the length of their ICU stay did not differ from other patients (26 +/- 30 vs 30+/- 26 days; *p *= 0.15)

Risk factors associated with mortality in univariate analysis are shown in Tables 2 and 3. At ICU admission the Charlson index, the modified IADL index, the number of organ failures and the SOFA score were associated with mortality; however, the ADL index was not. During the ICU stay the number of organ failures, the SOFA score, the need for mechanical ventilation or extra-renal support, sequentially assessed, were significantly associated with mortality. Interestingly, all patients (*n *= 22) needing extra-renal support after Day 3 died. In addition, the decision to activate care withdrawal, the length of the hospital stay and hospital re-admission were also associated with 3-month mortality.

**Table 2:** Risk factors associated with mortality at 3 months

Characteristics	Alive (*n *= 38)	Dead (*n *= 61)	Univariate analysis *P *value Odd Ratio [95% CI]	Multivariate analysis *P* value Odd Ratio [95% CI]
*ICU admission (n = 99)*				

Age	78.8 ± 3.1	79.7 ± 3.3	p = 0.18 1.09 [0.96-1.24]	

Male (%)	24(63.1%)	41(68.3%)	p = 0.68 1.20 [0.51-2.80]	

BMI, mean ± SD	28.3 ± 4.8	26.3 ± 6.5	p = 0.4 0.96 [0.88-1.05]	

Charlson index, median (IQR)	5(4-6)	7(5-8)	p = 0.003 1.45 [1.12-1.87]	p = 0.0025 1.6 [1.2-2.2]

ADL index, mean ± SD	5.4 ± 1.	5.5 ± 1.1	p = 0.36 1.31 [0.91-1.86]	

Cognitive score, mean ± SD	1.6 ± 1.3	1.0 ± 1.4	p = 0.03 0.73 [0.53-0.99]	

SAPS II score, median (IQR)	49(39-63)	55(41-70)	p = 0.16 1.01[0.99-1.04]	

APACHE II score, median (IQR)	24(16-28)	24(20-31)	p = 0.18 1.03 [0.99-1.08]	

SOFA score, median (IQR)	6(3-8)	7(5-11)	p = 0.035 1.13 [1.01-1.27	

Organ failures, mean ± SD	1 ± 1.1	1.7 ± 1.1	p = 0.003 1.77 [1.20-2.61]	p = 0.02 2.5 [1.15-5.2]

Mechanical ventilation, *n *(%)	20(52.6%)	42(68.9)	p = 0.16 0.5 [0.2-1.3]	

NIV, *n *(%)	14(36.8%)	17(27.9%)	p = 0.47 1.51 [0.58-3.95]	

Shock, *n *(%)	12(31.6%)	29(47.5%)	p = 0.18 1.96 [0.84-4.59]	

Extra-renal support, *n *(%)	2(5.2%)	10(16.3%)	p = 0.12 0.28 [0.04-1.53]	

BMI, body mass index; ADL, Activity of Daily Living; SAPS II score, Simplified Acute Physiologic Score II, APACHE II score, Acute Physiology and Chronic Health Evaluation; SOFA score, Sequential Organ Failure Assessment; NIV, non invasive ventilation; MV, mechanical ventilation.

**Table 3:** Risk factors during ICU stay and follow up after hospital discharge associated with mortality at 3 months

Characteristics	Alive (*n *= 38)	Dead (*n *= 61)	Univariate analysis *P *value; Odds Ratio [95% CI]
Day 3 (n = 77)^*^			

SOFA score, median (IQR)	3(2-5)	6(3-9)	p = 0.002; 1.26 [1.08-1.47]

Organ failures, mean ± SD	0.5 ± 0.7	1.4 ± 1.2	p = 0.002; 2.77 [1.48-5.19]

Mechanical ventilation, *n *(%)	12(32%)	37(61%)	p = 0.003; 0.21 [0.07-0.64]

NIV, *n *(%)	7(18%)	9(14%)	p = 0.8; 1.38 [0.39-4.86]

Shock, n (%)	3(8%)	15(25%)	p = 0.06; 0.25 [0.05-1.1]

Extra-renal support, *n *(%)	0	14	p = 0.008*; NA

Day 7 (n = 48)^**^			

SOFA score, median (IQR)	2(3-4)	5(4-8)	p = 0.04; 1.30 [1.01-1.67]

Organ failures, mean ± SD	0.3 ± 0.6	0.9 ± 1.0	p = 0.03; 2.86 [1.09-7.53]

Mechanical ventilation, *n *(%)	8(21%)	25(41%)	p = 0.04; 0.21 [0.05-0.094]

NIV, *n *(%)	3(8%)	2(3%)	p = 0.53; 3.11 [0.35-31.27]

Shock, n (%)	0	3	p = 0.29*; NA

Extra-renal support, *n *(%)	0	9	p = 0.012*; NA

*All ICU Stays (n = 99)*			

Mechanical ventilation, *n *(%)	21(55%)	47(77%)	p = 0.04; 0.37 [0.14-0.97]

NIV, *n *(%)	18(47%)	22(36%)	p = 0.37; 1.6 [0.64-3.98]

Shock, *n *(%)	13(34%)	37(61%)	p = 0.03; 0.34 [0.13-0.86]

Extra-renal support, *n *(%)	3(8%)	22(36%)	p = 0.003; 0.15 [0.03-0.61]

Duration of ventilation, median (IQR), days	5.2 ± 6.2	4.5 ± 9.5	p = 0.71; [0.95-1.04]

ICU length of stay, median (IQR), days	12.7 ± 18.9	16.2 ± 18.7	p = 0.38; 1.01 [0.99-1.035]

Decision to activate care withdrawal, n (%)	4(10%)	32(52%)	p = 0.001; 0.11 [0.03-0.37]

*After ICU discharge (n = 57)*			

Hospital length of stay	38.1 ± 29.6	23.6 ± 25.6	p = 0.002; 0.98 [0.97-0.99]

Hospital readmission post discharge	4 (10.5%)	6 (85.7%)	p = 0.001; 0.02 [0.002-0.21]

*9 and 13 patients discharged alive and dead from ICU, respectively at day 3

**21 and 30 patients discharged alive and dead from ICU, respectively at day 7

Sequential Organ Failure Assessment; NIV, non invasive ventilation; MV, mechanical ventilation. NA: Not applicable

* by Fisher exact test

In multivariate analysis only the Charlson index [Adjusted Odds Ratio, 1.6; 95% CI, 1.2-2.2; *p *< 0.0025] and the number of organ failures [Adjusted Odds Ratio, 2.5; 95% CI, 1.15-5.2; *p *< 0.02] at ICU admission were independently associated with short-term mortality.

### Physical Dependence, Cognitive Status, Subjective Health Status at 3-month Follow-up

Forty-five patients (45%) were discharged from hospital to domicile (*n *= 32), families (*n *= 2) or an institution (*n *= 11). At the 3-month follow-up, 10 patients were re-hospitalized: 3 patients had been admitted to the ICU and 6 had died. One patient was lost to follow-up. Therefore, at 3 months 38 patients (35%) were still alive.

Compared with pre-admission, the physical dependence and the cognitive status of survivors had changed very little at 3 months. The pre-admission ADL index compared to the 3-month ADL index (*n *= 36) was 5.5 **± **0.9 vs 4.3 ± 1.6 (*p *= 0.04), and the pre-admission cognitive score compared to the 3-month cognitive score (*n *= 36) was 1.1 **± **1.3 vs 2.9 ± 1.40 (*p *= 0.62). The assessment of subjective health status by the Nottingham Health Profile (NHP) score was obtained directly in 26 survivors (68%) at 3 months. Twelve patients with difficulties with language (*n *= 7), memory (*n *= 3) or hearing (*n *= 2) were unable to answer at the time of the phone interview at 3 months. However, these difficulties had been present in 4 of them before ICU admission. The mean NHP score was 213.1 **± **132.8 at 3 months. The social isolation score (26.2 ± 28.6) and the emotional reaction score (25.2 ± 26.9) were lower than other variables tested (sleep 37.7 ± 28.8, pain 38.9 ± 27.6, energy 42.5 ± 35 and physical mobility 42.7 ± 36.1).

## Discussion

In industrialized countries, the high number of older persons in need of intensive care is a common problem with ethical and social consequences [19]. The present study reports the short-term mortality in critically ill older patients under medical care (≥75 yrs) admitted to the ICU. In survivors, physical and cognitive dependence and subjective health status is also described. With a 3-month survival rate of 39%, this study argues that age itself should not be a reason for withholding ICU admission as previously reported [2]. In addition, at 3 months, most of the survivors lived independently with an acceptable quality of life. However, a high comorbidity level, the number of organ failures and the need for extra-renal support at the early phase of intensive care, were the most strongly associated factors for death. This result could have implications for early identification of geriatric patients for whom intensive treatment could be regarded as futile and for whom only palliative care should be provided.

### Baseline Characteristics

Few studies have focused on outcomes in the oldest patient population (≥ 75 yrs) admitted into an ICU [2,3,20-25]. Except for 1 study [24], all have included a mixed population: medical, unplanned surgical and planned surgical. In this report, we focus exclusively on critically ill older persons under medical care, the population associated with the highest mortality [3]. A series of 100 older persons (15% of our ICU population), consecutively admitted to the ICU, were included in the analysis. Among them, 39% were 80 years and older. This result was in accordance with previous reports focused on the oldest patients in the ICU, ≥ 70 yrs [6,26], ≥ 75 yrs [25], or ≥ 80 yrs [3,24], but differed from the 9% recently reported [27], suggesting a more restrictive admission policy in the latter. Despite a median Charlson index of 6 [IQR, 4 to 7] corresponding to a high comorbidity level, patients assessed by ADL and cognitive indices had a low physical and cognitive dependence level. In accordance with previous studies [6,26,27], more than half of the patients were independent and approximately 90% had been living at home before ICU admission, suggesting a selected population with good functional status. This result supports a recent study [2] reporting that functional status was an independent factor associated with refusal of ICU admission.

### Mortality

The 3-month mortality rate of 61% reported in this study did not differ from those previously reported in the oldest patients admitted to an ICU [2,3,20,24,26,27]. In accordance with previous studies, the majority of non survivors died during the ICU stay and half of them within the first week. Whether earlier treatment limitation decisions may influence this result is unlikely since the length of the ICU stay did not differ between patients with or without treatment limitations (26 +/- 30 vs 30+/- 26 days; *p *= 0.15), suggesting that these decisions were made late in the ICU stay. However, consistent with previous reports [28,29] focused on all ICU populations regardless of age, a decision to forgo life-sustaining therapy was associated with death. Nevertheless, information about the frequency and time of decisions to limit treatment is rarely described. In our practice, decisions are made by consensus among all the ICU staff (including physicians, nurses and consultants as needed) in accordance with the French "Leonetti" law regarding patient rights related to end of life. With the exception of conscious patients without cognitive impairment, patients and families are not involved in the decision-making process. However, their consent to follow the staff's decision is sought. Futility and poor expected quality of life are the most frequent reasons for withholding or withdrawing life-support therapies. Among studies focused on critically ill older persons, only 1 study [2] reported the proportion of patients (70%) subject to treatment withholding or withdrawal decisions. This report contrasts with the 36% treatment limitation decisions in our cohort.

### Predictors of Mortality

Consistent with previous studies [3,24-27], severe comorbidities and initial severity of illness are independently associated with short-term mortality.

Although the Charlson index was predictive for death in a large cohort of geriatric patients (≥75 yrs) hospitalized in medical wards consequent to emergencies [16], it has rarely been assessed as a predictor for death in critically ill older patients (≥ 75 yrs). However, regardless of age, previous reports identified the Charlson index as an independent factor associated with hospital mortality in a mixed population (ICU and intermediate ICU) [30] or after discharge from an intermediate-care unit [31]. This index was also reported as an important prognostic factor for long-term survival after ICU discharge in trauma patients [32] and a mixed population (medical and surgical) [33,34].

In addition, the occurrence or persistence of multi-organ failure concurrent with the need for extra-renal support after Day 3 was also strongly associated with death. Few studies have addressed the clinical impact of dialysis in critically ill elderly patients. Nevertheless, this result is consistent with 2 recent studies which reported hemofiltration [5] and dialysis results [35], respectively, as predictive factors for death in patients 70 years and older with abdominal pathologies and in mixed medical-surgical populations 80 years and older admitted to the ICU. In contrast, dialysis was not associated with mortality in older persons (≥ 70 yrs) hospitalized in the ICU for ≥ 30 days [6]. Differences in definitions of older persons, type of recruitment (medical, unplanned surgical and planned surgical) and variables studied may explain this difference.

With the aim of optimizing the balance between life-saving and non beneficial intensive care, we believe these data could help intensive care specialists decide whether or not continuation of intensive care is the treatment of choice.

Interestingly, in this setting the cognitive score but not the physical dependence index was associated with death. This result suggests that the ICU outcome in older persons could be more strongly influenced by impairment of cognitive functions than physical dependence. These findings are consistent with a previous study [36] reporting that the Instrumental Activity Daily Living index and moderate to severe cognitive impairment, assessed by the Short Portable Mental Status Questionnaire, is predictive of death. Our results also agree with other studies which failed to show an association between physical dependence, assessed by the ADL index, and death in the ICU's oldest patients (≥. 85 yrs) [20] and in older persons needing ventilatory support [26]. In contrast, the ADL index was reported as a predictor of poor long-term outcome in other studies [36,37]. Further research is needed to clarify the impact of physical dependence and cognitive function impairment on short-term mortality in an elderly population undergoing medical treatment in the ICU.

### Physical Dependence, Cognitive Status and Subjective Health Status in Survivors

Regarding the ADL and cognitive indices, there is little change in physical dependence and cognitive status in survivors at a 3-month follow-up. Only a transient decrease in physical status was observed, in accordance with previous studies [8,22]. In addition, subjective health status assessed by the NPH index was consistent with previous studies [6,38,39] using the same generic health indicator to assess quality of life in intensive care survivors. According to these reports, the psychosocial aspects of life (isolation and emotional reaction categories) were better than those of all other variables tested, in comparison with the results of the NPH index in the French general population of mixed age without hospitalization [15]. This result is also consistent with the accumulated body of literature [4] on the outcomes of older survivors of ICU stays, regardless of the choice and quality of tools used to assess quality of life. Nevertheless, these consistent results should be interpreted cautiously because of the small number of studies that specifically address this topic, the lack of a uniform approach to quality of life assessment and difficulties in follow-up after ICU discharge that make comparisons between series of patients challenging. In addition, the oldest patients could have a more positive perception of their quality of life than younger patients due to more acceptance of their physical limitations [39].

### Limits

This study has some limitations. The mono-centric design of the study, the relatively small sample size, the absence of assessment of subjective health status of patients before ICU admission, as well as the fact that during the period of study 70 older persons (≥ 75 yrs) requiring medical care were withheld from the ICU, may limit the interpretation and relevance of our data. Addressing the latter, the proportion of older persons who were not admitted to the ICU is consistent with a recent report [2], and in our clinical practice triage decisions regarding admission to the ICU require the opinion of 2 senior practitioners and are guided by the recommendations of the Society of Critical Care Medicine [40]. We believe that this report contributes useful information about clinical outcomes, predictors of death and long-term quality of life in a selected older population requiring intensive care. Firstly, our study focuses on a population at high risk of ICU death (42% in our cohort vs 29% in patients 65 to 74 years old and 21% in patients 64 years old and younger during the same period, data not shown). Moreover, the study includes a high proportion (39%) of older persons 80 years and older. Finally, we used the most commonly employed scoring systems (specifically the Charlson index and the ADL index) available for geriatric populations.

## Conclusion

In a selected population of older persons (≥ 75 yrs) under medical care, admission into the ICU is associated with a 3-month survival rate of 38% with little impact on physical and cognitive dependence and subjective health status. Nevertheless, a high comorbidity level (ie, Charlson index), multi-organ failure and the need for extra-renal support at the early phase of intensive care, could be considered as predictors of death. Further research is needed to improve the knowledge required to optimize the balance between life-saving and non beneficial intensive care in the most elderly patient population.

## Abbreviations

ADL: Activity of Daily Living; APACHE II score: Acute Physiology and Chronic Health Evaluation; BMI: body mass index; MV: mechanical ventilation; NIV: non invasive ventilation; SAPS II score: Simplified Acute Physiologic Score II; SOFA score: Sequential Organ Failure Assessment.

## Competing interests

The authors declare that they have no competing interests.

## Authors' contributions

CD and SC initiated the study, and the design. CD and SC were responsible for data collection during ICU stay. After ICU discharge, the follow up was conducted by SC. CG, SC and CD performed the statistical analysis and were involved in the interpretation of the results. CD and SC wrote the manuscript, and JJP and PC helped to draft the manuscript. AS, XV, FP, NT, MR and DDC, contributed to the conception and design of the study and revision of the manuscript. All authors read and approved the final manuscript.

## Authors' information

This work was presented in part at the annual congress of the Société de Réanimation de Langue Française (SRLF) held in January 2008, Paris, France.
